# Pancreatic Fibrosis (Early Chronic Pancreatitis) as Emerging Diagnosis in Structural Causes of Dyspepsia: Evidence from Endoscopic Ultrasonography and Shear Wave Elastography

**DOI:** 10.3390/diagnostics11071252

**Published:** 2021-07-13

**Authors:** Chung-Tsui Huang, Tzong-Hsi Lee, Cheng-Kuan Lin, Chao-Yi Chen, Yi-Feng Yang, Yao-Jen Liang

**Affiliations:** 1 Graduate Institute of Applied Science and Engineering (ASE), College of Science and Engineering, Fu Jen Catholic University, No. 510, Zhongzheng Rd., Xinzhuang Dist., New Taipei City 242062, Taiwan; 0989206131ng@gmail.com (C.-T.H.); g730325@hotmail.com (C.-Y.C.); endlessjustin@hotmail.com (Y.-F.Y.); 2 Department of Internal Medicine, Division of Gasteroenterology and Hepatology, Far Eastern Memorial Hospital, No. 21, Sec. 2, Nanya S. Rd., Banciao Dist., New Taipei City 220, Taiwan; thleekimo@yahoo.com.tw (T.-H.L.); widelin@mail.femh.org.tw (C.-K.L.)

**Keywords:** early chronic pancreatitis, pancreatic fibrosis, endoscopic ultrasonography, chromogranin-A, dyspepsia

## Abstract

A new concept for the diagnosis and management of non-functional dyspepsia in guidelines was lacking in the past decade. Medical advancement has proven pancreatic fibrosis (essential image evidence of early chronic pancreatitis) to be a cause of dyspepsia and related to pancreatic exocrine dysfunction. This study aimed to analyze the clinical picture, biomarker, and percentage of pancreatic fibrosis in the dyspeptic population. A total of 141 consecutive patients were retrospectively enrolled. They were diagnosed with peptic ulcer disease, 9.2% (*n* = 13); pancreatic fibrosis, 17% (*n* = 24); pure Helicobacter pylori infection, 19.9% (*n* = 28); functional dyspepsia, 53.2% (*n* = 75); and chronic pancreatitis, 0.7% (*n* = 1). Among those with pancreatic fibrosis, (*n* = 24), 11 were diagnosed on the basis of a pancreatic acoustic radiation force impulse exceeding 1.4 m/s, and the remaining 13 were diagnosed with early chronic pancreatitis with at least three of the Japanese endoscopic ultrasonography criteria. The anatomic distribution of parenchymal criteria of early chronic pancreatitis was head, 53%; body, 38%; and tail, 9%. There were 17 cases (71%, 17/24) without Helicobacter pylori and whose dyspepsia improved after pancreatic enzyme replacement with a ratio of 82.3% (14/17). Of the 141 cases, 19 received gastric emptying scintigraphy and Western blot analysis of chromogranin-A in duodenal mucosa. Delayed gastric emptying was more common in functional dyspepsia and chromogranin-A was expressed more in pancreatic fibrosis. In conclusion, pancreatic fibrosis (including early chronic pancreatitis) outnumbered peptic ulcer disease in the dyspeptic population and pancreatic enzyme therapy was effective for 82% of cases. In early chronic pancreatitis, pancreatic fibrosis is dominant in the head location, and duodenum mucosa chromogranin-A is a potential biomarker with increased expression in an age-matched manner.

## 1. Introduction

Dyspepsia refers to upper abdomen discomfort or indigestion with a prevalence in approximately 20% of the general population [1,2]. It can be etiologically categorized as functional or organic, and around 20% of all causes are attributable to the structural pathogenesis of digestive organs [1,3]. According to Rome IV criteria, there are two types of dyspeptic symptoms: epigastric pain syndrome and postprandial distress syndrome [4]. Peptic ulcer disease is traditionally regarded as a leading cause of organic dyspepsia [1,5]. However, the incidence of peptic ulcer disease has been decreasing in recent decades, probably due to the use of acid suppressive agents with gastric mucosa protection effects and aggressive eradication of Helicobacter pylori, an etiology for peptic ulcers [6,7]. Therefore, theoretically, other diagnoses regarding the structural causes of dyspepsia will increase.

Chronic pancreatitis is a progressive fibro-inflammatory disease and conventionally diagnosed at the end stage, with global prevalence ranging from 5 to 12 per 100,000 population-years and 44.5 per 100,000 persons from the Japan surveillance data in 2016 [8,9]. The overall prevalence of chronic pancreatitis is about 50/100,000 persons worldwide [10]. The causal relationship between exocrine dysfunction of chronic pancreatitis and non-ulcer dyspepsia was studied about three to four decades ago by analyzing the duodenal juice after injection of a Lundh test meal [11,12]. With advancements in medical technology such as endoscopic ultrasonography (EUS) and ultrasound shear wave elastography, it is easier to evaluate the early stage of chronic pancreatitis [13,14,15,16,17,18]. A disease entity, early chronic pancreatitis, has gradually been acknowledged under international consensus [10,19,20,21,22]. The diagnosis of early chronic pancreatitis requires the first essential factor of pancreatic fibrosis and then is supported by symptoms, risk factors, and biomarkers [16,19]. Pancreatic fibrosis can be proved non-histologically by various image modalities [16]. The term “pancreatic fibrosis” is mostly used in elastography, computed tomography, and magnetic resonance imaging examination; otherwise, the term “early chronic pancreatitis” is usually used in the EUS procedure to diagnose morphologic changes of pancreatic fibrosis [16]. The risk factors of early chronic pancreatitis are similar to those of chronic pancreatitis because both share the same spectrum of diseases [21,23,24]. According to a literature review for the diagnostic biomarker in chronic pancreatitis, a suitable biomarker for early-stage chronic pancreatitis is lacking. A large-scale research platform has been established in the United States of America for the discovery of a high-quality biomarker [25].

In 2018, there were two milestone studies about early-stage chronic pancreatitis in dyspepsia. One was a 213-case prospective study proving that EUS-diagnosed chronic pancreatitis accounted for 21% of dyspeptic patients presenting epigastric pain syndrome. Among the chronic pancreatitis group, 60% of patients had early-stage chronic pancreatitis [26]. The other study enrolled 100 consecutive patients with functional dyspepsia and showed that approximately 24% were attributable to EUS-diagnosed early chronic pancreatitis [27]. However, these two studies lacked data on the overlap between Helicobacter pylori and early chronic pancreatitis.

The infection rate of Helicobacter pylori is important for analyzing the dyspepsia population as per guideline recommendations [28]. Thus, the primary purpose of this study is to diagnose the percentage of each structural cause of dyspepsia, namely, pancreatic fibrosis (including early chronic pancreatitis), peptic ulcer disease, and Helicobacter pylori infection in the dyspeptic population. Consecutive patients were recruited from communities complaining of chronic dyspepsia with epigastric pain syndrome or postprandial distress syndrome after excluding cases with typical symptoms of gastroesophageal reflux disease (GERD).

The second purpose is to compare gastric emptying between pancreatic fibrosis (including early chronic pancreatitis) and functional dyspepsia in a small case series. Impaired gastric emptying is usually noted in functional dyspepsia and studies are ongoing [29,30,31]. However, the percentage of delayed gastric emptying in pancreatic fibrosis (including early chronic pancreatitis) remains unknown. In this small case series, the duodenal first-part mucosa was obtained for Western blot analysis of chromogranin-A, which is a protein secreted by enteroendocrine cells and used as their surrogate [32,33]. It is hypothesized that pancreatic exocrine dysfunction in pancreatic fibrosis (including early chronic pancreatitis) would feed back to the duodenal enteroendocrine cells to enhance pancreatic enzyme secretion by upregulating the secretion of signal molecules. This hypothesis was evidenced in a small case series consisting of non-alcoholic fatty pancreas dyspeptic patients with and without EUS-diagnosed early chronic pancreatitis. The early chronic pancreatitis group showed higher expression of duodenal mucosa chromogranin-A [34]. Whether this phenomenon is consistent in the more heterogeneous dyspeptic patients is evaluated in this study.

## 2. Methods

Between Jan 2019 and Apr 2020, consecutive patients who agreed to receive upper gastrointestinal endoscopy (Olympus Co., Tokyo, Japan) and trans-abdomen sonography (Siemens Acuson S2000 Ultrasound System, Munich, Germany) for the investigation of chronic dyspepsia were retrospectively surveyed. Patients were recruited from the digestive clinic at Far Eastern Memorial Hospital. The inclusion criteria were age between 20 and 70 years, and chronic dyspepsia with symptoms of epigastric pain syndrome or postprandial distress syndrome. The exclusion criteria were symptoms of gastro-esophageal reflux disease, alarming symptoms or signs (weight loss, overt anemia, overt gastrointestinal bleeding, and dysphagia), cirrhosis, end-stage renal disease or uremic syndrome, patient refusal to participate in the study, high risk of endoscopic procedure in pregnant woman, malignancy, and suspected cancer.

The investigated factors of chronic dyspepsia included pancreatic acoustic radiation force impulse (ARFI; Siemens Acuson S2000 Ultrasound System, Munich, Germany), surveillance of Helicobacter pylori by histology or rapid urease test, and significant organic etiologies. Upon consent of the patients, EUS (GF-UE 260; Olympus Co., Tokyo, Japan) was performed if pancreatic ARFI > 1.4 m/s or in the presence of risk factors for early chronic pancreatitis, followed by gastric emptying scintigraphy scan and Western blot analysis of chromogranin-A, cholecystokinin, and glucagon-like peptide-1 in the four pieces of tissue biopsied in the duodenum first-part mucosa.

**Figure 1:**
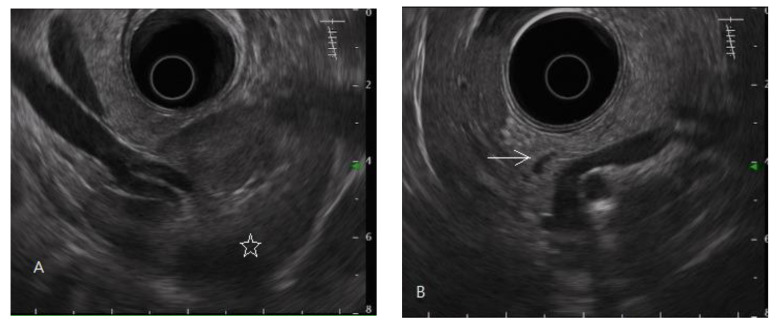

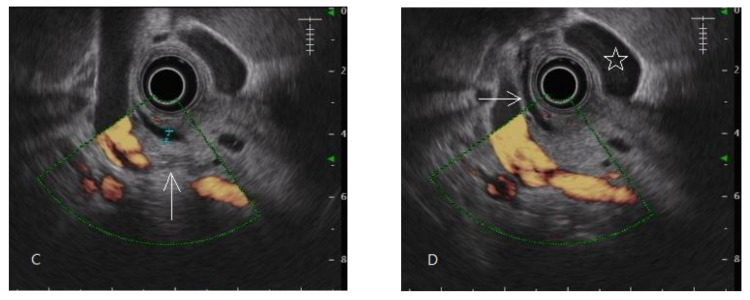
Endoscopic ultrasonography of a normal pancreas. The normal pancreatic parenchyma is homogeneous, and the main pancreatic duct edge is non-hyperechoic. (**A**) Pancreatic tail, body; spleen (star mark), splenic vessels. (**B**) Pancreatic body, neck, partial main pancreatic duct (arrow mark). (**C**) Pancreatic head, uncinate process (hypoechoic ventral part, arrow mark); gallbladder; partial main pancreatic duct. (**D**) Pancreatic head; gallbladder (star mark); main pancreatic duct (arrow mark).

**Figure 2:**
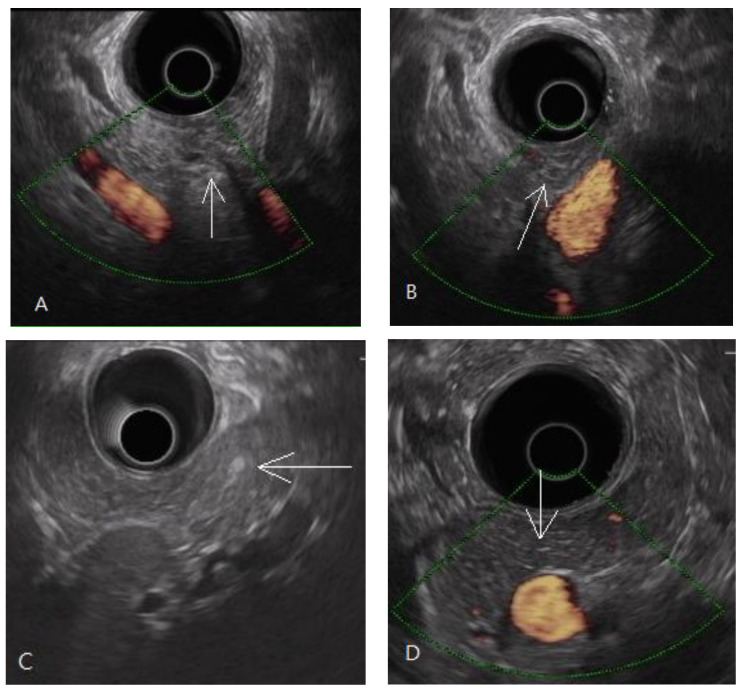
Endoscopic ultrasonography of early chronic pancreatitis. (**A**,**B**) Pancreatic head lobularity (arrow mark) showing heterogeneous parenchyma with hyper/hypo echoic areas mixing, irregular hyperechoic stranding. (**C**) A hyperechoic focus without an acoustic shadow (arrow mark) in the pancreatic tail. (**D**) Hyperechoic stranding in body, neck (arrow mark).

In this study, the diagnosis of pancreatic fibrosis was pancreatic ARFI value > 1.4 m/s (mean of 10 measurements in pancreatic head or body) [16] or meeting the EUS diagnostic criteria of early chronic pancreatitis. EUS-diagnosed early chronic pancreatitis has at least three of the seven total factors of the EUS criteria for early chronic pancreatitis proposed by the Japan Pancreas Society (JPS). The seven factors are lobularity with honeycombing, lobularity without honeycombing, hyperechoic foci without shadowing, stranding, cysts, dilated side branches, and hyperechoic main pancreatic duct margin [35], illustrated by normal and abnormal images in Figure 1 and Figure 2, respectively.

The sonographic classification of fatty liver is mild (grade 1), with higher hepatic echogenicity than the renal cortex; moderate (grade 2), with hepatic vessel blurring; and severe (grade 3), with obscured vision of hepatic vessels [36]. The sonographic classification of fatty pancreas is mild (grade 1), with increased echogenicity of the pancreatic body similar to the echogenicity of retroperitoneal adipose tissue; moderate (grade 2), with a blurred outline of the main pancreatic duct; and severe (grade 3), with an invisible splenic vein and deeper structures [37].

### 2.1. Western Blot Analysis

Duodenal mucosa proteins were extracted by homogenization of tissue in protein extraction solution (iNtRON Biotechnology) and 1 mm zirconia beads. The solution was subsequently managed by five 3 min shocks and centrifugation at 13,000 rpm for 20 min. The supernatants were collected, and protein concentrations were estimated using the BCA protein assay kit (Thermo). The 30 μg protein was resolved in SDS-polyacrylamide gels on a Minigel apparatus and transferred to a PVDF membrane using a semidry transfer cell. The transblotted membrane was washed 3 times and blocked with PBST containing 5% nonfat milk for 60 min, followed by incubation with the appropriate primary antibody. The membrane was washed 3 times by PBST. Finally, the membrane was probed with horseradish peroxidase-conjugated secondary antibody and visualized by enhanced chemiluminescence. The density of the blot was further analyzed by densitometry using the TotalLab TL120v2009. The primary antibodies used were antichromogranin-A (Abcam, ab199014), CCK8 (Biorbyt, orb10260), GLP-1 (Biorbyt, orb10719), and β-actin (Santa Cruz, sc-47778). For analysis of protein expression, the case with the lowest pancreatic ARFI value in the non-pancreatic fibrosis group was defined as 1.0. The other cases were presented as a relative expression value for statistical calculation.

### 2.2. Statistical Analysis

The numerical variables were presented as median or percentage. The categorical variables were presented as percentage. For group data, χ2 and Pearson tests were used for categorical and continuous variables, respectively. An independent samples *t* test was used for paired data. Data analyses were performed using a standard software package (SPSS version 20.0, Chicago, IL, USA). A *p*-value of < 0.05 was considered statistically significant.

## 3. Results

**Figure 3:**
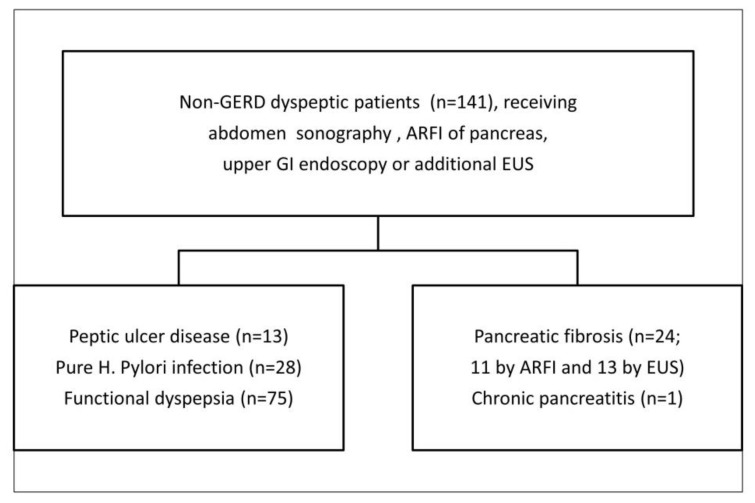
The numbers of each subgroup, GERD: gastro-esophageal reflux disease, GI: gastrointestinal, EUS: endoscopic ultrasonography, ARFI: acoustic radiation force impulse, H. pylori: Helicobacter pylori.

A total of 144 consecutive dyspeptic patients who agreed to undergo the upper gastrointestinal endoscopy and abdominal sonography with ARFI evaluation of pancreas were surveyed. Three cases were excluded because of hepatic solid hypoechoic tumor, pancreatic solid tumor, and gastric B-cell lymphoma. Among the 141 enrolled patients, 13 had EUS-diagnosed early chronic pancreatitis, 11 had pancreatic fibrosis diagnosed by pancreatic ARFI > 1.4 m/s, and 1 had typical sonographic findings of chronic calcified pancreatitis. Moreover, 75 cases had functional dyspepsia without Helicobacter pylori infection, 28 had pure infection of H. pylori without peptic ulcer, and 13 had peptic ulcer disease (7 patients with duodenal ulcer, 5 patients with gastric ulcer, and 1 patient with gastric and duodenal ulcers simultaneously). The demography and flowchart of each subgroup are summarized in Table 1 and Figure 3, respectively.

In the pancreatic fibrosis (containing early chronic pancreatitis) subgroup (*n* = 24), the risk factors included alcoholic drinking, smoking, hyper-triglyceridemia with history of acute pancreatitis, type 2 diabetes mellitus, and gallstones [23]; only three cases were without an overt risk factor.

**Figure 4:**
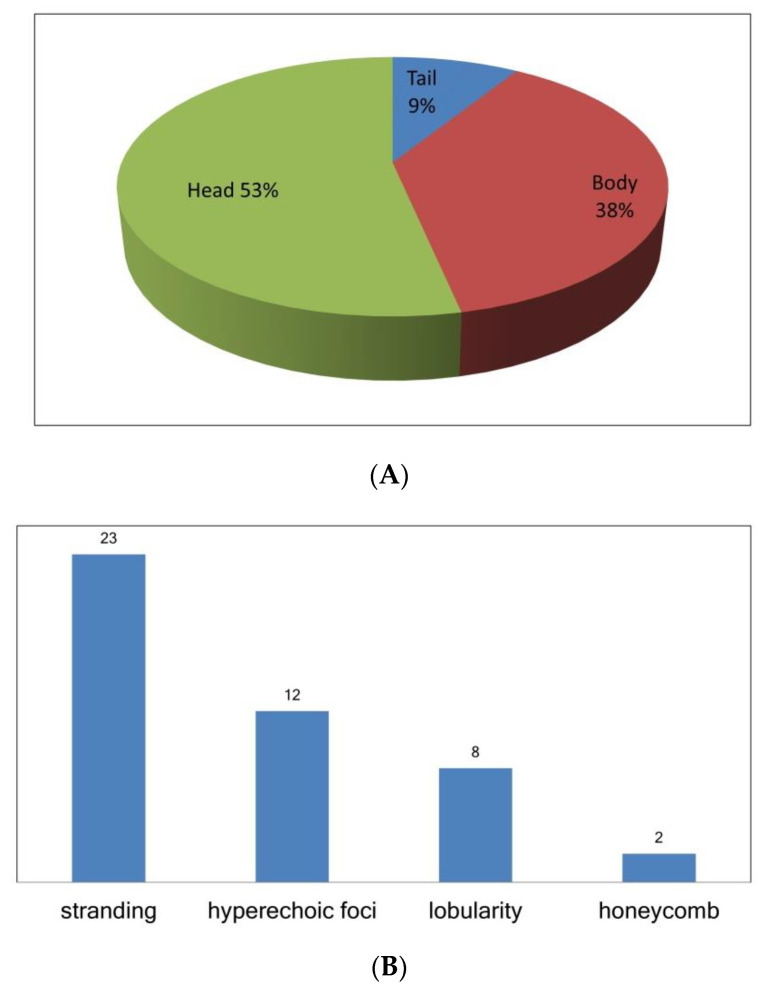
A total of 45 counts of the 4 EUS criteria of pancreatic parenchymal fibrosis in the 13 patients with EUS-diagnosed early chronic pancreatitis; (**A**) the ratio of EUS criteria counts in pancreatic head (including neck, uncinate process), body, and tail; and (**B**) the counts of each EUS parenchyma criteria for pancreatic fibrosis. EUS: endoscopic ultrasonography.

Among the 13 patients with EUS-diagnosed early chronic pancreatitis, there were altogether 45 counts of the four criteria of pancreatic parenchymal fibrosis: lobularity with honeycombing (*n* = 2), lobularity without honeycombing (*n* = 8), hyperechoic foci without shadowing (*n* = 12), and stranding (*n* = 23). According to pancreatic anatomic separation, the ratios of these four criteria in different locations were 53% in the head (including neck, and uncinate process), 38% in the body, and 9% in the tail, as shown in Figure 4.

Among the 141 enrolled patients, there was a series of 19 patients with informed consent to undergo a gastric emptying scintigraphy scan and Western blot analysis of chromogranin-A, cholecystokinin, and glucagon-like peptide-1 in duodenal first-part mucosa. There was 1 case with duodenal ulcer, 5 cases with EUS-diagnosed early chronic pancreatitis, and 13 cases with functional dyspepsia. The gastric emptying was more delayed in the functional dyspepsia group compared with the pancreatic fibrosis group (38.5% vs. 20%, p-value: 0.04), and chromogranin-A expression in duodenal mucosa was increased in the pancreatic fibrotic group with age-matched comparison (p-value: 0.04) Western blot analysis of the other two proteins, cholecystokinin and glucagon-like peptide 1, showed no significant difference between the pancreatic fibrotic and functional dyspepsia groups.

## 4. Discussion

**Figure 5:**
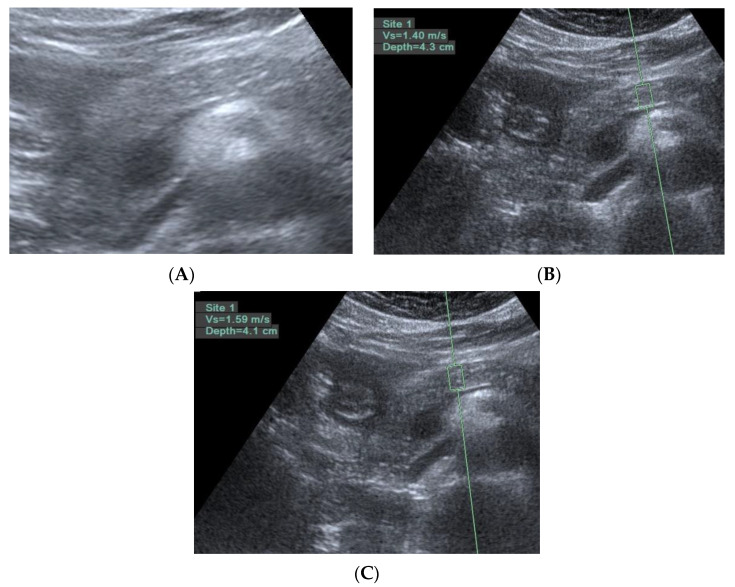
Three trans-abdomen sonographic images of the pancreas in the same patient. (**A**) Some hyperechoic foci in the pancreas; (**B**) if there are no hyperechoic foci in the region of interest, the pancreatic ARFI is 1.4 m/s; and (**C**) if there are hyperechoic foci in the region of interest, the pancreatic ARFI is higher (1.59 > 1.4 m/s).

In this study on the dyspeptic population, early chronic pancreatitis was defined as pathologically equivalent to pancreatic fibrosis. Pancreatic fibrosis is a term for image diagnosis conventionally used in shear wave elastography, computed tomography, and magnetic resonance imaging [17,38]. Early chronic pancreatitis is a diagnostic term for the EUS procedure first introduced in the nomenclature by a review article published in 1995 [39]. A prospective study using EUS to diagnose alcoholic early chronic pancreatitis was published in 2002 [13]. In the past two decades, early chronic pancreatitis has mostly been diagnosed by EUS in studies [14,15,21,40,41,42,43,44,45]. By definition, early chronic pancreatitis is a syndrome requiring symptoms, risk factors, biomarkers, and image diagnosis, according to an international consensus [19]. The EUS criteria for diagnosing early chronic pancreatitis or chronic pancreatitis were proven to be histologically related to pancreatic fibrosis with approximately 80% accuracy [46,47]. The fibrotic change in the pancreas was focal occurrence [46]. It was evidenced in the distribution of the EUS diagnostic criteria for early chronic pancreatitis, as shown in Figure 2. There was also evidence of focal fibrosis in the pancreatic ARFI procedure, as shown in Figure 5.

Pancreatic fibrosis (including early chronic pancreatitis) accounted for 17% of the whole dyspeptic population in this study of 141 community patients with non-GERD chronic dyspepsia willing to seek medical help. This ratio is reasonable compared to the data of two milestone articles mentioned in the Introduction section. On average, functional dyspepsia accounts for 80% of dyspepsia [1,28]. Early chronic pancreatitis theoretically accounts for at least 19.6% of the whole dyspeptic population, because the study performed in Japan showed that approximately 24.5% of functional dyspepsia is early chronic pancreatitis [27]. Another study performed in Spain showed that the percentage of early-stage chronic pancreatitis, with three or four criteria of EUS for chronic pancreatitis, was 15.9% of the whole dyspeptic population, as seen in Figure 1 of that article (34/213 cases) [26].

In the dyspeptic population, the percentage of pancreatic fibrosis (including early chronic pancreatitis) outnumbered that of peptic ulcer disease in this study and another study [26]. This entity, known as early chronic pancreatitis or pancreatic fibrosis, is clinically meaningful because pancreatic exocrine dysfunction was detected in 79.4% of patients by the endoscopic pancreatic function test or secretin-enhanced magnetic resonance image in [26]. The approach to the management of dyspepsia is consistent, with no new concepts in the last decade. The three factors required for it to be managed are endoscopic surveillance, a test for Helicobacter pylori, and a proton pump inhibitor trial, all of which appear in the three guidelines published in 2010, 2017, and 2020 [1,3,28]. It is time to consider pancreatic fibrosis (including early chronic pancreatitis) as an emerging diagnosis of structural causes of dyspepsia, with a percentage of between 15.9 and 19.6% [26,27]. In the current study, approximately 29% of the cases were pancreatic fibrosis (including early chronic pancreatitis) simultaneously infected by Helicobacter pylori. The infection of Helicobacter pylori is the treatment priority in the management of dyspepsia [1,48]. Then, it is reasonable to survey pancreatic fibrosis in non-ulcer dyspeptic patients after the eradication of Helicobacter pylori. Among the 24 patients with pancreatic fibrosis in this study, seven had Helicobacter pylori infection (29.2%) and received eradication therapy. Pancreatic enzyme replacement was administered to the other 17 patients without Helicobacter pylori infection and with 82.3% (14/17) positive therapeutic response.

For those patients with progressive pancreatic fibrosis and risk factors for pancreatic adenocarcinoma, EUS-guided fine-needle biopsy for fibrotic tissue acquisition can be considered. The pancreatic fibrotic tissue can be analyzed for pathology, KRAS mutation, and other molecular diagnoses.

Current techniques for diagnosing pancreatic fibrosis (including early chronic pancreatitis) have two demerits. First, the trans-abdomen shear wave elastography is interfered by central obesity, exemplified by a case in the current study and who eventually underwent EUS to diagnose early chronic pancreatitis. Second, EUS is an invasive procedure with inter-observer variability, showing a kappa value (level of agreement) of about 0.6–0.8 [15,18,49]. The optimal resolution is probably the EUS technique combined with shear wave measurement, which objectively and quantitatively assesses the stiffness of pancreatic parenchyma [50,51].

The exocrine secretion of the pancreas is controlled by the vagal nerve and signaling protein molecules released by enteroendocrine cells [52,53]. Between pancreatic fibrosis and functional dyspepsia in the present case series, duodenal mucosa chromogranin-A, a surrogate of enteroendocrine cells, had significantly higher expression in the pancreatic fibrosis group by age-matched comparison, but not cholecystokinin or glucagon-like peptide-1. This phenomenon reflects that enteroendocrine cells existed more in the pancreatic fibrosis group and the peptide hormone was not elevated in the fasting state. The presence of more enteroendocrine cells probably means they were preparing for the production of more secretagogues for pancreatic fibrosis (early chronic pancreatitis), which has proven to have a 79% chance of inadequate exocrine function [26]. Another point is that the significantly higher expression of chromogranin-A was dependent on age-matched comparison. This phenomenon implicates quantitative variation in enteroendocrine cells with age.

## 5. Conclusions

In conclusion, pancreatic fibrosis (including early chronic pancreatitis) is an emerging diagnosis in structural causes of dyspeptic patients, and pancreatic enzyme replacement can be tried with an approximately 82% response rate. The fibrotic change is predominant in the pancreatic head location, a phenomenon similar to pancreatic adenocarcinoma. Duodenum mucosa chromogranin-A is a potential biomarker for pancreatic fibrosis with increased expression.

## Figures and Tables

**Table 1:** The demography of the subgroups of the dyspeptic population.

Characteristics	Pancreatic Fibrosis (Early Chronic Pancreatitis) *n*= 24	Peptic Ulcer Disease *n* = 13	Helicobacter Pylori Infection *n* = 28	Functional Dyspepsia *n* = 75
Percentage of the 141-case group	17%	9.2%	19.9%	53.2%
Female/male	15/9	4/9	13/15	42/33
Age (median)	53.5	56	57	43
BMI (median)	24.9	24.5	24.5	23.7
H. pylori (%)	29.2	DU 87.5 GU 50	100	0
Gallstone Cholecystectomy (%)	14.3 0	15.4 0	7.1 0	5.3 1.3
Fatty liver (≥grade 2) (%)	25	7.7	17.9	8
Fatty pancreas (≥grade 2) (%)	66.7	15.4	14.3	17.3
Type 2 DM (%)	20.1	15.4	25	9.3

BMI: body mass index, H. pylori: Helicobacter pylori, DM: diabetes mellitus, DU: duodenal ulcer, GU: gastric ulcer.
